# Multiple scattering in observations of the GPM dual‐frequency precipitation radar: Evidence and impact on retrievals

**DOI:** 10.1002/2014JD022866

**Published:** 2015-05-04

**Authors:** A. Battaglia, S. Tanelli, K. Mroz, F. Tridon

**Affiliations:** ^1^ National Center Earth ObservationUniversity of LeicesterLeicesterUK; ^2^ Earth Observation Science, Department of Physics and AstronomyUniversity of LeicesterLeicesterUK; ^3^ Jet Propulsion LaboratoryCalifornia Institute of TechnologyPasadenaCaliforniaUSA

**Keywords:** radar, GPM‐DPR, multiple scattering, graupel

## Abstract

This paper illustrates how multiple scattering signatures affect Global Precipitation Measuring (GPM) Mission Dual‐Frequency Precipitation Radar (DPR) Ku and Ka band reflectivity measurements and how they are consistent with prelaunch assessments based on theoretical considerations and confirmed by airborne observations. In particular, in the presence of deep convection, certain characteristics of the dual‐wavelength reflectivity profiles cannot be explained with single scattering, whereas they are readily explained by multiple‐scattering theory. Examples of such signatures are the absence of surface reflectivity peaks and anomalously small reflectivity slopes in the lower troposphere. These findings are relevant for DPR‐based rainfall retrievals and stratiform/convective classification algorithms when dealing with deep convective regions. A path to refining the rainfall inversion problem is proposed by adopting a methodology based on a forward operator which accounts for multiple scattering. A retrieval algorithm based on this methodology is applied to a case study over Africa, and it is compared to the standard DPR products obtained with the at‐launch version of the standard algorithms.

## Key Points

- GPM‐DPR observations are affected by multiple scattering in deep convection
- The multiple‐scattering tail can be used for better characterizing the ice phase
- Multiple‐scattering effects can be included in DPR retrievals

## 1 Introduction

The NASA‐Japan Aerospace Exploration Agency Global Precipitation Measurement (GPM) [*Hou et al.*, 2013] core satellite has been successfully launched in February 2014. The GPM core satellite is equipped with a dual‐frequency precipitation radar (DPR) operating at *K*_u_ (13.6 GHz) and *K*_a_ (35.5 GHz) band and with a multifrequency microwave radiometer (GMI). The overarching GPM goal is to advance the understanding of the global water/energy cycle variability through more frequent and accurate measurements of precipitation. Thanks to its dual‐frequency capability relative to the single‐frequency (Ku band) radar used in Tropical rainfall Measuring Mission (TRMM) [*Kummerow et al.*, 1998] the DPR is expected to improve our knowledge of precipitation processes with more detailed information on microphysics and better accuracies in rainfall retrievals. Deep convective storms which represent the archetype source of precipitation are the focus of this paper. Their importance stems from the fact that they are responsible for most of the extreme weather events, they play a key role in the exchange between the upper troposphere and the lower stratosphere, and they are associated with high rain rates and huge amounts of latent heat release. Thanks to the possibility of combining high‐resolution (250 m vertical resolution at nadir) radar reflectivity profiles with IR, passive microwave, and lightning data the TRMM mission has provided unprecedented information on the global distribution of intense convective storms and of their structure [e.g., *Zipser et al.*, 2006; *Liu and Zipser*, 2013; *Liu et al.*, 2008].

It is therefore timely to investigate what is the added value of the Ka radar channel when observing deep convection. Spaceborne radar observations of convective cores are particularly challenging for at least two reasons (see also discussion in *Battaglia et al.* [2011, 2013]). First, they often exhibit 3‐D structure variability at spatial scales smaller than the DPR instrument footprint (4 × 4 km^2^) with consequential nonuniform beam filling issues [*Takahashi et al.*, 2006; *Iguchi et al.*, 2009; *Tanelli et al.*, 2012; *Meneghini et al.*, 2012]. Second, they are characterized by strong attenuation, which can drive the signal below the sensitivity threshold despite the high effective reflectivities. Correcting for attenuation is a thorny process, especially when no integral constraints to the total attenuation are available [*Meneghini and Liao*, 2013; *Kubota et al.*, 2014]. Things become even more difficult because attenuation can be partly compensated by multiple‐scattering (MS) enhancement (see review by *Battaglia et al.* [2010]). This is particularly true at Ka band where large ice particles can be considered almost perfect scatterers (that is, they cause significant extinction, but contrary to liquid raindrops, this is due almost entirely to scattering and not absorption). The detection of MS enhancement and its quantification are quite difficult if only one frequency is available. In the case of CloudSat's W band Cloud Profiling Radar MS effects had been predicted before launch [*Battaglia et al.*, 2007], were confirmed postlaunch [*Battaglia and Simmer*, 2008; *Battaglia et al.*, 2010], and were accounted for in the official precipitation products [*Haynes et al.*, 2009] to the extent p ossible with single‐frequency observations and their associated ambiguities. In the case of TRMM evidence to expect significant MS in TRMM/Precipitation Radar (PR) in extreme cases was provided only in *Battaglia et al.* [2014]. The presence on the DPR of a second frequency (the Ku channel), which is much less attenuated, provides an unprecedented opportunity to check the physical consistency between models and observations and to assess the relevance of MS effects following the approach adopted for High‐Altitude Imaging Wind and Rain Airborne Profiler airborne observations [*Battaglia et al.*, 2014]. In that work the presence of anomalously small slopes of the observed radar backscatter profile at Ka with respect to Ku throughout a layer of several kilometers close to the surface (where the opposite is expected to occur due to rain attenuation) was explained in an Occam's razor approach via MS theory. The resulting observable feature is a characteristic “knee” when considering the difference (ratio) between the Ku‐ and Ka‐measured reflectivities (*Z*_att_) expressed in logarithmic (linear) units, known as dual‐wavelength ratio (DWR) and defined as

(1) $$\text{DWR}(z)[\text{dB}]\equiv 10\;\underset{10}{\log}\frac{Z_{\text{att},K_{u}}(z)[{\text{mm}}^{6}\;m^{-3}]}{Z_{\text{att},K_{a}}(z)[{\text{mm}}^{6}\;m^{-3}]}=Z_{\text{att},K_{u}}(z)[\text{dB}Z]-Z_{\text{att},K_{a}}(z)[\text{dB}Z],$$

where *z* is the altitude. In this paper similar evidence of MS for the DPR is documented for a convective case study over Africa. Data are presented in section 2 with a discussion of its salient features and its explanation in terms of MS theory. A retrieval algorithm based on optimal estimation which accounts for MS is applied to the case study to demonstrate the added value of the Ka observations. Conclusions are finally drawn in section 3.

## 2 A Case Study

**Figure 1:**
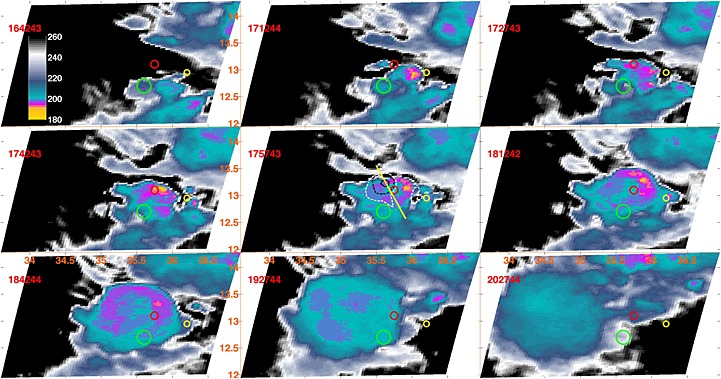
Evolution of the storm as seen by MSG‐IR (acquisition time shown in red). The red circle marks the position of the main convective core of interest at the time of DPR overpass, the yellow marks a nearby younger cell, and the green marks the area of stratiform rain produced by the anvil. In the middle, corresponding to the closest time to the overpass, the contour of 160 K for the GMI brightness temperature at 37V, 89V, and 166V GHz are indicated in continuous black, dashed black, and continuous white, respectively. The yellow line corresponds to the satellite ground track.

**Figure 2:**
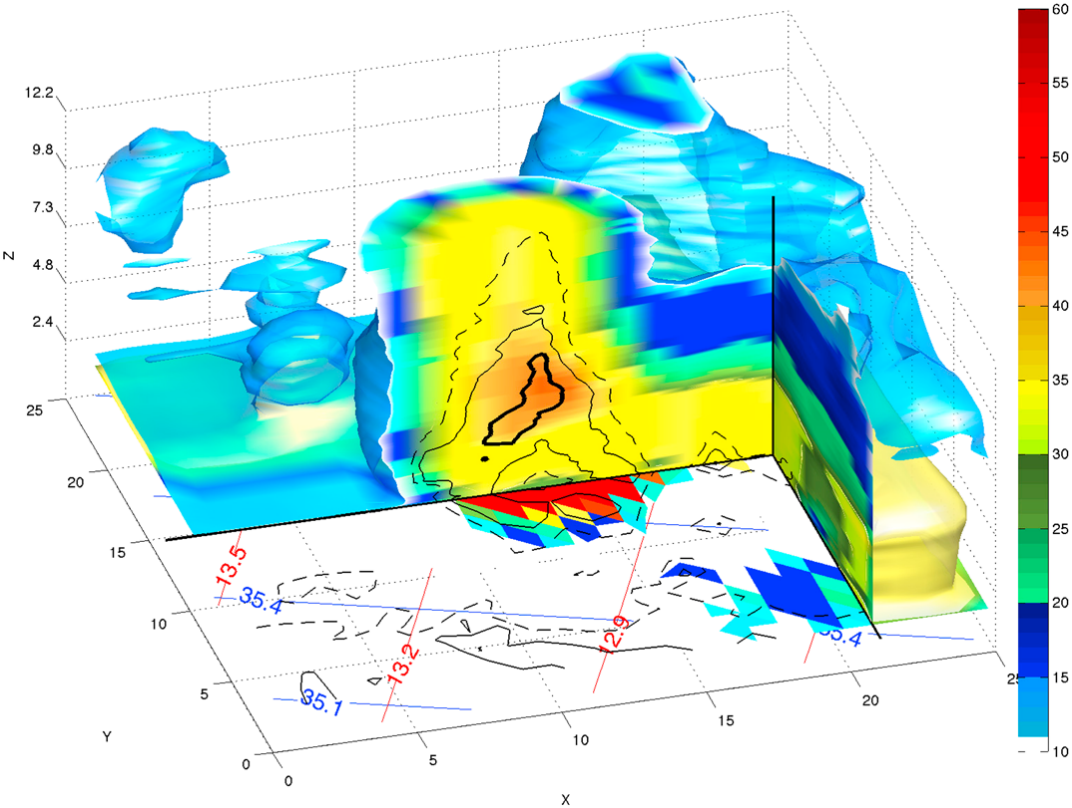
Three‐dimensional view of the convective event. The 3‐D shape (surfaces) corresponds to the measured Ku band 12 and 30 dB*Z* reflectivity factors in blue and yellow, respectively. The vertical sections and sections at 12 km altitude also show measured reflectivities in dB*Z* at Ku band (see color bar). The black dashed and thin and thick solid contour lines correspond to 10, 16, and 22 dB DWR levels, respectively. The image shows such contour lines on the vertical section and at the surface for near‐surface values. The color map at the surface shows the Ku band path‐integrated attenuation (PIA) in dB (doubled). Blue and red texts indicate the latitude and longitude coordinates.

The convective cell selected in this paper to represent a vast range of convective cores was observed by DPR on 9 September 2014 at 17:58*Z* over 13.1^∘^N, 35.75^∘^E, the eastern edge of the clay plains in Sudan north of the Blue Nile and downstream of the Ethiopian Plateau. Geostationary imagery from Meteosat Second Generation (MSG) in Figure 1 shows a fairly typical evolution of this particular cell following the diurnal amplification of a wave: as of 16:42*Z* the initial signatures of convective activity are evident immediately to the SE of the location where the cell will be eventually observed by DPR (marked by the red circle). The image closest to the DPR overpass is the middle one, where also the GMI 160 K contour lines for three of the channels are shown for reference. The GPM core satellite was descending from N‐NW to S‐SE (Figure 1, yellow line in the middle). The general spread of the DPR markers, IR imagery, and GMI brightness temperatures is a reminder of the importance of parallax for this type of phenomenon: the correct location of the cell, in this case, is best approximated by the DPR marker since the GPM had an almost direct overpass over it and the core was not affected by significant vertical shear (see Figure 2), GMI is affected by a shift to the NW by its conical scanning geometry, and MSG is shifted to the E‐NE by its position to the west and over the equator.

**Figure 3:**
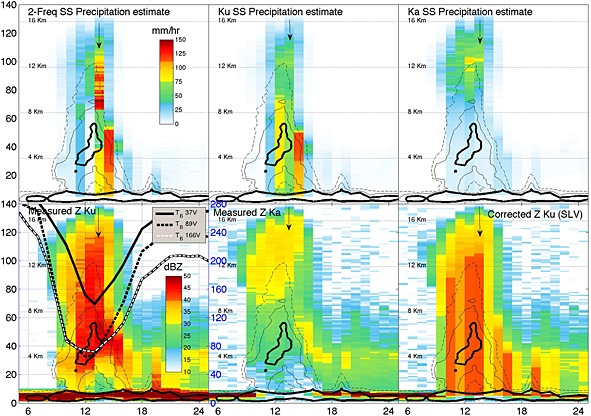
Along‐track cross section of the convective system shown in Figure 2 with (bottom left) Ku‐ and (bottom middle) Ka‐measured vertical profiles. *X* and *y* axes correspond to along‐track and vertical DPR bins, respectively. Observed brightness temperatures for three GMI channels as indicated in the legend (shifted by four footprints along track) are also superimposed in Figure 3 (bottom left). (bottom right) The retrieved (attenuation‐corrected) Ku band reflectivity and the profiles of retrieved rain rates with (top right) Ka only, (top middle) Ku only, and (top left) Ku + Ka are derived from the Level 2 V03B‐GPM products.

By 20:27*Z* no fresh convective activity is visible in MSG imagery, and following images (not shown here) show the dissipating stages of the anvil until it moves out of the area and fades away by 03:00*Z* on 10 September. While technically a mesoscale convective system, this was neither a particularly strong nor long‐lived one. A depiction of the storm as captured by DPR is shown in Figure 2 in a virtual view from the west (note the red and blue longitude and latitude markers) and in Figure 3 in a complete cross section along the nadir beam of DPR. The 30 dB*Z* level of the main convective core reached 16.5 km altitude, and the 40 dB*Z* reached 14.5 km. The maximum height of the Ku 40 dB*Z* echo is often used as a proxy for the strength of convection [*Zipser et al.*, 2006]. According to Figure 2 in *Zipser et al.* [2006], this corresponds to a class of precipitation features that occur 0.01% of the time. The Ka band channel lost completely the surface return (Figure 3, bottom middle), with the Ku band channel exhibiting a two‐way attenuation of 15.7, 22.9, and 27.7 dB in the twelfth, thirteenth, and fourteenth pixels, respectively. These and the brightness temperature signatures (down to 131, 56.5, and 69.1 K for the 37V, 89V, and 166V channels, respectively) are clear indicators of intense convection and graupel (or small hail) production, with instantaneous precipitation rates in excess of 50 mm/h but less than 150 mm/h (more discussion later in regard to specific estimations). Most of the profiles at the center of the convective tower (identified by the arrow in Figure 3) are identified by the GPM classification module as convective.

A second, younger cell is visible to the E‐SE (Figure 1, marked by a yellow circle), and a region of stratiform rain under the anvil associated with the general convective activity is visible to the south (Figure 1, marked by a green circle). Notably, no precipitation was detected in the area to the west of the main cell, contrary to what one may infer from IR‐based retrievals applied to the MSG imagery or from the parallax‐affected GMI brightness temperature values. The feature that we focus on in this paper is marked in Figures 2 and 3 by the three black contour lines: the thick solid contour marks the region where the DWR exceeded 22 dB, and the thin solid and dashed contours mark the 16 and 10 dB levels, respectively.

As discussed in *Battaglia et al.* [2014] and summarized in section 1, in the presence of significant extinction, such as in this case, all single‐scattering models predict that DWR will, for the most part, monotonically increase as one moves from the top of the cloud to the surface. This is because DWR changes due to differential reflectivity signatures in the Mie region are of smaller magnitude than those due to extinction, which is always larger at Ka band than at Ku band. On the contrary, the observed region of very high DWR at midlevel above an area where the DWR decreases approaching the surface cannot be explained by single‐scattering theory: this is the predicted occurrence of what we refer to as the “DWR knee” in DPR measurements, due to the MS signatures generated by the dense scattering ice aloft. The impact of MS on the standard at‐launch DPR retrieval algorithm [*Kubota et al.*, 2014] is evident in Figure 3: as it was the case for TRMM and for CloudSat, at‐launch retrieval algorithms do not include the impact of MS for practical reasons (a choice dictated mainly by the necessity to implement computationally efficient algorithms and the lack of actual data to fine‐tune more complex solutions until the mission is launched). Therefore, they attempt to “explain” the observed reflectivity profiles according to the classical solutions of the weather radar equation at dual frequency, which simply do not include in their forward model anything that can reproduce the actual observations. The result is quite evident in Figure 3 (top left) the precipitation rate retrievals by the two‐frequency algorithm exhibit unrealistic variations horizontally and vertically in the profiles at the center of this core. They do, however, produce reflectivity profiles corrected for attenuation (bottom right) that appear possible. This apparent contradiction is actually not entirely unexpected because of the ambiguous nature of the retrieval process: the solver produces a solution that is the least distant from observations, and in the space of the corrected reflectivities, it is in fact plausible. But to achieve that, within the internal constraints and relationships built in under single‐scattering assumption, it is forced to adopt unphysical configurations of the precipitating particles. Notably, the heritage solution from TRMM/PR, which uses only Ku band data (top middle), delivers retrievals that while probably also affected by uncertainties larger than the norm, they are more accurate and fall in a physically plausible range and morphology. This is because the Ku band channel is not affected by MS as much as Ka, and the intrinsic a priori constraints are derived from 15 years of climatological analysis and TRMM validation experience: they may not be always as accurate as for more manageable precipitation configurations, but their overall accuracy is generally well established. Finally, the Ka‐only retrievals, as expected, are of limited value in this type of precipitation because of the extreme extinction. Should we therefore conclude that the Ka band signal is only detrimental and should be discarded? This would be a practical and efficient solution as the final product for DPR could simply detect this situation and discard the dual‐frequency solution to fall back to the still respectable accuracy level of TRMM/PR. In the rest of this paper, however, we will try to convey how Ka band does in fact carry very useful information that can indeed improve the accuracy of the Ku‐only product and should be considered for future improvements in the GPM/DPR retrieval algorithm.

### 2.1 Salient Features in the Observed Profiles

**Figure 4:**
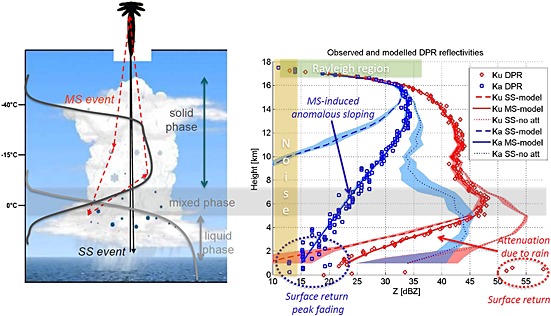
(left) Schematic for the MS mechanism in a convective cloud. (right) Measured and modeled DPR reflectivities for the profile indicated by the black arrow in Figure 3. Observed (modeled) profiles are plotted with symbols (lines). Red and blue colors correspond to the Ku and Ka bands, respectively.

We now focus at the center of the convective core: the profiles marked by the arrow in Figure 3 are shown in Figure 4 (red diamond and blue squares for Ku and Ka, respectively). As a reference, we have produced on the left side of the figure a cartoon of the scene under observation with a sketch of the expected profiles of solid and liquid phase hydrometeor contents.

The Ku band reflectivity profile follows a behavior already observed by TRMM, with the presence of a characteristic well‐pronounced nose in correspondence of the mixed‐phase region (roughly corresponding to the grey‐shaded horizontal band) and a strong decrease of reflectivities in the bottom part of the profile, a clear signature of heavy rain. If the TRMM *Z*‐*R* and *k*‐*Z* relationships adopted for rain in convective regions [*Iguchi et al.*, 2000, Table 1] are used, 2.5 dB/km of one‐way attenuation (like that experienced in the area highlighted by the red arrow) roughly corresponds to 60 mm/h. Note that the surface signal is well emerging from the atmospheric return at around 500 m (red ellipse). Turning to the Ka profile (blue diamonds) while in the upper part (roughly above 12 km) seems to follow the expected pattern with reflectivities first almost identical to the Ku reflectivities (Rayleigh region), and then increasingly smaller due to Mie effects, the rest of it presents some not entirely unexpected surprises [*Battaglia and Simmer*, 2008; *Battaglia et al.*, 2010, 2014].

1. There is no nose in correspondence to where mixed‐phase hydrometeors are expected to be (Feature 1). It is also interesting to note that there is no change in the sloping of the Ka profile at 11 km where the Ku profile presents a bump.
2. The sloping of the Ka reflectivity profile is completely anomalous when compared to that of the Ku channel (Feature 2, blue arrow). In the specific case, the averaged extinction coefficient is about 1.7 dB/km, i.e., even smaller than the extinction coefficient measured by the Ku radar. If the *k*‐*R* relationship proposed by *Matrosov et al.* [2006] (*R*[mm/h] = 3.6*k*[dB/km]) is used, a rainfall rate of 6 mm/h is retrieved, a value far below what was predicted at Ku.
3. There is no clear discontinuity in the reflectivity profile in correspondence to the surface as highlighted by the blue dashed circle (Feature 3).

**Figure 5:**
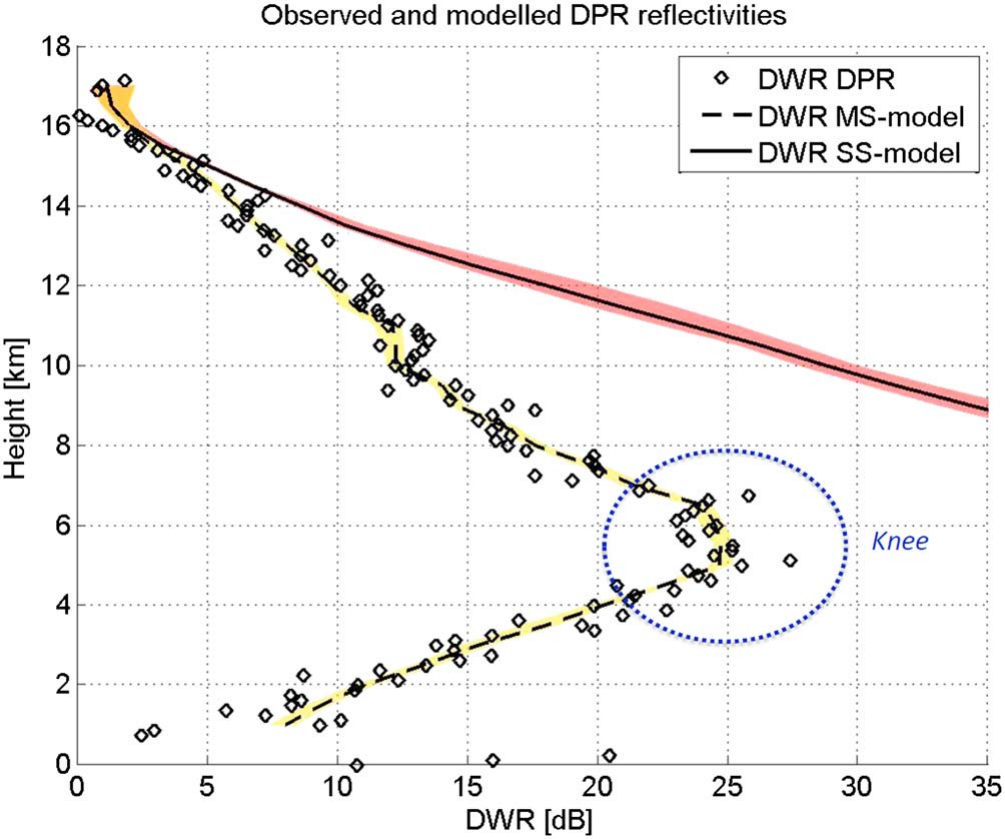
Same as in Figure 4 but for measured and modeled dual‐wavelength ratios.

The plot of the DWR for the profile shown in Figure 4 is depicted in Figure 5. The presence of Feature 2 is the driver for the appearance of the DWR knee (dotted ellipse). It is important to note the considerable size of the knee in terms of both extent (from 6 km all the way down to the near surface) and depth (∼20 dB excursion). These characteristics are completely different from those predicted by single‐scattering theory and well depicted in Figure 2 of *Le and Chandrasekar* [2013] for stratiform and convective systems. Only a small bump would be expected in correspondence of the melting zone. Vice versa the measured DWR profile presents features similar to those already reported in Ku‐Ka airborne observations [*Battaglia et al.*, 2014, Figure 2].

### 2.2 Explanation of the Knee With Multiple‐Scattering Effects

The interpretation of the DWR knee is straightforward if MS effects are included. Several theoretical [*Kobayashi et al.*, 2005; *Battaglia et al.*, 2006] and observational [*Battaglia and Simmer*, 2008; *Battaglia et al.*, 2008; 2014] studies have demonstrated that when dealing with millimetric spaceborne radar observation of strongly scattering media, the radar signal may have significant contributions from second and successive orders of scattering. In fact, at millimeter frequencies, attenuation is driven by scattering more than absorption processes, especially when dealing with ice phase hydrometeors. This means that the radiation emitted by the radar is not absorbed and consequently lost but actually keeps propagating inside the medium. If the footprint of the instrument is comparable to the mean free radiation path—defined as the inverse of the extinction coefficient—MS contributions may become important [*Kobayashi et al.*, 2007; *Battaglia et al.*, 2010].

**Figure 6:**
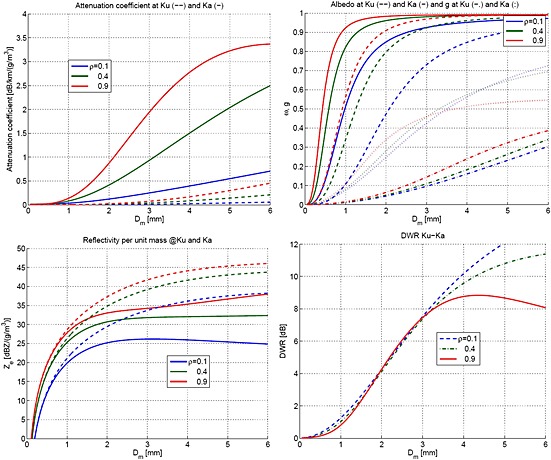
(top left) Attenuation coefficients per unit mass at *K*_*u*_ (dashed lines) and *K*_*a*_ (continuous lines) as a function of the mean mass‐weighted diameter, *D*_*m*_, for exponential size distributions. Different colors correspond to different ice densities as indicated in the legend. (top right) The same for the single‐scattering albedo, *ω* (dashed and continuous lines), and asymmetry parameter, *g* (dash‐dotted and dotted lines). (bottom) The same (left) for the effective reflectivity per unit mass and (right) for the DWR.

Some of the single‐scattering properties of ice particles exponentially distributed and with densities equal to 0.1, 0.4, and 0.9 g/cm^3^ are plotted for the two DPR frequencies in Figure 6. We will refer for convenience to these three densities as “snow,” “graupel,” and “hail,” respectively. When considering Figure 6 (top left), it is clear that ice is not producing significant attenuation at Ku band (dashed lines), while it may have a significant impact at Ka (continuous lines). A 1 g/m^3^ graupel layer with a mean mass‐weighted diameter of 3 mm can produce a one‐way attenuation at Ka band of 1 dB/km (and this number linearly increases with the equivalent water content). Most of such attenuation is indeed producing scattering because the single‐scattering albedo for such a cloud is equal to 0.98 (top right). The corresponding scattering mean free path is equal to 4.3 km, i.e., comparable with the GPM‐DPR footprint. The effect is significantly stronger (weaker) for hail (snow).

Another aspect is worth noting: unlike what happens at visible wavelengths, at millimeter wavelengths hydrometeors diffuse radiation with a predominance of wide‐ versus small‐angle‐scattering events [*Hogan and Battaglia*, 2008]. This is caused by the behavior of the hydrometeors scattering phase function which is not as highly forward peaked as at visible wavelengths [e.g., see *Bohren and Huffman*, 1983]. For instance, at Ka band asymmetry parameter values rarely exceed values of 0.6 (Figure 6, top right). As a consequence, a large portion of radiation is deflected at almost right angles and therefore dwells in an environment which is completely different from that experienced by radiation undergoing single‐scattering events but corresponding to the same apparent range from the radar. In the cartoon on the left side of Figure 4 while the radiation following the red path (third order of scattering) undergoes scattering events only within the ice and mixed phase, the radiation following the black path (single scattering) passes through the whole ice layer and is backscattered by the rain underneath. If heavy rainfall is present, the single‐scattering contribution can be strongly attenuated so that only higher orders of scattering, which are not affected by rain attenuation because they do not go through it, can significantly contribute to the received power at the given apparent range. In such a situation a MS tail emerges at apparent ranges which correspond to the rain layer. With this in mind, the interpretation of the previously described Feature 2 is straightforward: the anomalous sloping of the Ka reflectivity in the lower layers has nothing to do with the actual rain present there, but it is induced by MS pulse stretching caused by the ice‐scattering layer in the upper levels. The fact that even the mixed‐phase layer enhancement is completely lost in the Ka signal (Feature 1) suggests that the MS tail emerges well above the rain layer. The layer between 12 and 16 km is likely to be a significant source of MS. This is corroborated by the Ku‐ and Ka‐measured reflectivities reaching in that region values up to 34 and 43 dB*Z*, respectively (Figure 4). When using the reflectivities per unit mass at Ku band depicted in Figure 6 (bottom left), it is possible to show (not shown) that for all the three densities here considered Ka attenuation coefficients of at least 1 (2) dB/km are produced when the Ku reflectivities are exceeding 40 (43) dB*Z* in correspondence to *D*_*m*_ values ranging between 3 and 5 mm. Even larger values of Ka attenuation result by assuming smaller *D*_*m*_. On the contrary the Ka profile does not show a rapid decrease compatible with these values. This means that the medium is optically thick enough to cause MS within the DPR footprint, which partially compensates for attenuation. This conclusion is also corroborated (Figure 3, bottom left) by the strong brightness temperature depressions measured by the GMI, with the 37, 89, and 166 GHz reaching down 140, 68, and 70 K, respectively. Finally, Feature 3 observed in the Ka observed profile is also easily explained: the total attenuation is so large that the surface signal disappears below the MS tail, a phenomenon that is frequently observed in the CloudSat 94 GHz radar profiles [*Battaglia and Simmer*, 2008]. In such conditions path‐integrated attenuation (PIA) estimates based on the surface reference technique [*Meneghini et al.*, 2004; *Seto and Iguchi*, 2007] will be erroneous. In fact, the received signal in correspondence to the surface range (which is caused by the MS tail) certainly exceeds the actual surface return. As a result, estimates of the PIA will underestimate its true value, as already observed in CloudSat observations [*Battaglia et al.*, 2008]. For the Ku channel on the other hand, there is a well‐defined peak in correspondence to the surface (Figure 4, red ellipse), and the surface reference technique is applicable, though nonuniform beam filling issue can seriously affect its value [*Takahashi et al.*, 2006; *Tanelli et al.*, 2012].

### 2.3 Possible Retrieved Solutions

In order to get a better insight into this case study, a retrieval algorithm which accounts for MS has been developed. The retrieval is based on an optimal estimation technique [*Rodgers*, 2000], following similar approaches proposed in the past. It assimilates multifrequency reflectivity profiles and PIA measurements as detailed in *L'Ecuyer and Stephens* [2002] and *Grecu et al.* [2011]. Note that PIAs are included only if considered reliable by the GPM algorithm as identified by a value of 1 of the *PIAreliabFlag* variable. We briefly summarize the key components of the retrieval algorithm.

*Forward Model.* The code developed in *Hogan and Battaglia* [2008] is adopted as forward operator for computing the reflectivity profiles, the PIAs, and, via the perturbation method, the relevant Jacobians. This is a fast, approximate method for the calculation of the time‐dependent multiple‐scattering returns from radar based on the time‐dependent two‐stream approximation. The model is *O*(*N* ^2^) efficient or better for an *N*‐point profile, making it particularly suitable for use as the forward model in spaceborne radar retrieval schemes. While the code includes MS effects, three‐dimensional/nonuniform beam filling effects cannot be properly accounted for since the code is inherently one dimensional. As a result, a horizontally uniformly distributed atmosphere is assumed in the following analysis.

*Vector of Unknowns.* The algorithm retrieves profiles of equivalent water content (WC) of rain, frozen hydrometeors and cloud liquid water, mean particle sizes (*D*_*m*_) for rain and ice, and mean particle density for ice (*ρ*_ice_). The particle size distributions (PSDs) are assumed to be *Γ* functions with *μ* = 3 like in the GPM retrieval algorithm. As a result, PSDs are only functions of two parameters. We describe them in terms of the equivalent water content (WC) and the mean mass‐weighted diameter (*D*_*m*_), which have a simple physical interpretation.

*A Priori.* More and more research [e.g., *Grecu et al.*, 2011] is clearly pointing out the deficiencies of many of the ice phase assumptions embedded in retrieval algorithms, in particular in convective cores where retrievals are particularly challenging due to an increased range in the possible ice densities caused by the possible occurrence of graupel or hail, to the presence of occasionally large amounts of cloud liquid water, and to the large spatial variability of the PSD. It is therefore utterly difficult to define a meaningful set of a priori assumptions for the scene under observation. In such conditions it is also clear that a dual‐wavelength radar estimation method does not incorporate enough constraints to fully retrieve the ice microphysics, be the scene affected by multiple scattering or not. In that respect the role played by multiple scattering is twofold: on one side it undoubtedly complicates the interpretation of the signal; on the other side the correct fitting of the pulse‐stretching tail adds extra information about the ice layer only. Contrary to conventional algorithms, we have therefore decided to explore the possible space of solutions by adopting different a priori profiles based on different ice densities (ranging from 0.1 to 0.9 g/cm^3^), different mean diameters (compatible with the ice densities, e.g., graupel‐like particles having *D*_*m*_ not larger than 4 mm) and different liquid‐to‐solid transition heights. Rain (ice) is assumed to be present only below (above) a certain height, with a layer of coexistence between the two. High‐density ice (hail) is allowed to be present down to the surface level. Supercooled cloud is assumed to be present only above the freezing and below the −40^∘^C level. Each a priori profile is composed of an expectation and a standard deviation on such expectation used as measure of uncertainty in the iterations based on the Gauss‐Newton method for finding the solutions [*Rodgers*, 2000]. The idea is to investigate whether the GPM measurements are capable to narrow down significantly the solution space without attempting to achieve a unique solution.

**Figure 7:**
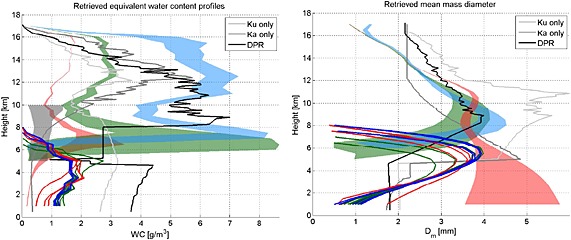
Retrieved profiles of (left) equivalent water content and (right) mean mass‐weighted diameter for the profile shown in Figure 4. Blue, green, and red colors correspond to snow, graupel, and hail a priori assumptions for the ice density with shaded areas corresponding to the ice phase and continuous lines to the liquid phase. Cloud water contents are also retrieved, and their variability is indicated by the grey‐shaded area. Black lines correspond to the results of the Level 2 GPM algorithms as indicated in the legend.

Each a priori profile can either converge to a solution (i.e., when the forward operator is capable to reproduce the observables within the preset tolerance) or generate a failed retrieval attempt. The information we are seeking lies in the presence of attractors that are common to multiple a priori conditions and in the absence of solutions for certain a priori conditions. This particular case study is compatible with all three ice densities. The most realistic solutions (i.e., with total equivalent water content lower than 10 g/m^3^ at all ranges) are shown in Figure 7. Blue, green, and red colors correspond to assuming the ice density of snow, graupel, and hail, while the grey and black curves are the V03B‐DPR products. In regard to the retrievals of ice aloft (between 9 and 16 km altitude), it is evident that the MS‐based retrieval of water content is very sensitive to the a priori assumption on ice density. This is qualitatively expected from the results shown in Figure 6 and the associated text: in order to achieve the necessary backscattering and scattering cross sections, larger amounts of low‐density ice are necessary to produce the same observables as low amounts of high‐density ice. In this regard, the DPR retrievals are relatively consistent among each other because they share similar a priori assumption on ice density. It is important to note that even when adopting a priori density profiles of 0.1 g/cm^3^ (constant for the whole height), the solutions tend to move the ice densities toward larger values (up to 0.21 g/cm^3^ and staying above 0.15 g/cm^3^ down to 10.5 km). This is a sign that the optimal estimation was attracted to higher‐density ice than the initial guess. Having permitted only a limited excursion on such parameter (because we aimed at simulating the a priori assumption that it would be low‐density snow), this was almost as high as the retrieved density was permitted to go. The higher densities on the other hand do not depart more than 10% from their initial values. Low densities (blue curves) tend to produce high value of equivalent water contents, in line with what is produced by the Level 2 GPM‐DPR algorithm (black lines) [*Iguchi et al.*, 2010]. On the contrary, the retrievals of mean particle size in the same layer are almost insensitive to ice density assumption as expected, and they are nicely in agreement with the DPR dual‐frequency estimate in the region between 9 and 12 km altitude. The Ku‐only and Ka‐only “retrievals” of particle size in this region are obviously driven only by the a priori assumptions internal to those single‐frequency retrievals. We note that above 12 km altitude the DPR retrievals exhibit a behavior that is probably due to first‐order assumptions (that are likely to be revised in the next version of the postlaunch algorithm): the retrieved diameter at 16 km altitude is 2.2 mm despite an observed DWR of 0 dB (see Figure 5). This particular aspect is not relevant to the focus of this paper.

In regard to the rain portion of the profile it is important to notice that, contrary to the single‐scattering retrievals, the multiple‐scattering retrieval seems to be quite robust in estimating rain water content and drop size (all a priori assumptions converge to somewhat similar solutions). While we have no means to validate that such retrievals are correct in this case study, this outcome is an encouraging result: apparently, the algorithm had a robust assessment of the MS contribution by the ice aloft (whatever its particular density may be) and could therefore always converge to the same solution. On the contrary, the single‐scattering‐based retrievals exhibit extreme and not realistic variability because they are trying to make sense of the observables without the proper forward operator, and therefore, it is only natural that the retrievals are driven by unintended effects of the a priori constraints and algorithmic solutions inside the solver.

Note that the decrease in the rain profile close to the surface is not believed to be a robust feature, being mainly caused by the constraint imposed by the two‐way Ku‐PIA (estimated to be equal to 22.9 dB from the SRT Level 2 product). However, given the structure of the system (Figure 2), the presence of non uniform beam filling effects is possible, and as a consequence, the PIA may be underestimated. As discussed hereafter the Ka is not providing any information in the rain layer, and as a result, the retrieval below 5 km is affected by uncertainties similar to the TRMM‐PR retrievals and will not be discussed further.

GMI brightness temperature observations can be used to test the plausibility of the retrieved solutions. Because of the marked 3‐D structure of the observed precipitation and of the previously mentioned parallax issues, we use the GMI radiometric observations only qualitatively (though they could be included directly in the retrieval algorithm). Forward modeled brightness temperatures computed by an Eddington approximation code [*Kummerow*, 1993] in correspondence to the retrieved profiles shown in Figure 7 produce brightness temperature depressions (not shown in the figure) which are deeper for higher ice densities. The simulated pairs of brightness temperature at 89 and 166 GHz are 108 and 115 K for the snow assumption, 85 and 91 K for the graupel assumption, and 73 and 83 K for the hail assumption. Therefore, high‐density particles (red and green colors) produce depressions more in line with GMI observations (compare with values reported in Figure 3, bottom left). It is clear that the suite of observations collected by the GPM core satellite pinpoints at the presence of heavily rimed aggregates or denser particles aloft, which is supported by climatological in situ observations of deep tropical convection [e.g., *Heymsfield et al.*, 2005]. In this case study, the radiometer is able to further reduce the degrees of freedom of the solution (i.e., it eliminates the low‐density solutions) left unresolved by the radar (which, we shall not forget, had eliminated a large number of degrees of freedom by providing a finite set of vertical profiles compatible with the radar observations). In summary, the unique combination of DPR reflectivities and GMI brightness temperatures provides an assessment of the ice layer aloft with much more confidence than ever achieved before (e.g., with TRMM or CloudSat retrievals).

In regard to the rain retrievals, since all radar solutions predicted mean rain rates below 5 km between 26 and 39 mm/h, the addition of these two radiometric channels does not significantly reduce the spread of the viable solutions.

The mean of the forward modeled reflectivities corresponding to all possible retrieved microphysical profiles are plotted in Figure 4 as continuous lines, while the shaded red/blue regions cover the variability of the different solutions. For illustration purpose, the single‐scattering forward reflectivities (dashed lines) and the nonattenuated single‐scattering forward reflectivities (dotted lines) with their corresponding envelopes are added as well. All the retrieved solutions consistently identify 15 km as the onset of MS effects for the Ka channel (i.e., where the dashed line starts significantly departing from the continuous line). Shortly below that altitude, contributions from second and successive orders of scattering dwarf the single scattering, which drops below the noise level already at about 9 km (where the dashed line is cut). On the other hand, the measured reflectivity remains above the noise level all the way down to the surface despite that the retrieved Ka one‐way PIA exceeds 70 dB. From the study of the Jacobians employed in the optimal estimation, it is possible to infer that the Ka radiation is not sensing any of the hydrometeors below roughly 6.5 km, i.e., Ka cannot retrieve any information about the rain layer. In that region all information derivable from the DPR comes from the Ku channel. Interestingly also, the Ku channel is slightly affected by MS in the layer closest to the surface.

## 3 Summary and Conclusions

This paper presents the first striking evidence of MS effects in the GPM‐DPR observations of a deep convective cell observed over Africa. Such effects were predicted before the launch of the spacecraft based on theoretical computations and on airborne observations [*Battaglia et al.*, 2014]. While it is basically impossible to reconcile the observed Ku and Ka band profiles with the use of single‐scattering theory (as adopted in the at‐launch DPR algorithm [*Kubota et al.*, 2014]), MS effects offer an easy explanation for the observations here presented.

There are some important consequences of this study for the GPM‐DPR Level 2 algorithms when dealing with measurements in *deep convection*.

1. Any dual‐frequency retrieval algorithm must account for MS. Fast forward codes are currently available and can be easily incorporated in 1‐D solution methodologies as shown in this work. If not, results are very likely totally wrong. In such situations it would be preferable to revert to Ku‐only products. However, as demonstrated in this work, the Ka channel and the MS tail can be fully exploited to better characterize the ice layer with great potential in sizing the ice particles. Additional information coming from the high‐frequency GMI channels has the potential to discriminate between different solutions based on different ice density assumptions which are compatible with DPR profiles. Since the Ka signal is not able to penetrate through the ice, only the Ku channel can be used to profile the rain underneath. Furthermore, even the Ku signal can be contaminated by MS enhancement in the pixels closest to the surface.
2. Surface reference techniques cannot be applied to derive PIA when MS contributions are overwhelming in correspondence to surface ranges. Such estimates of PIAs will be certainly underestimated by an unknown—arbitrarily large—amount.
3. Stratiform/convective precipitation‐type classification methods must also be upgraded according to the novel‐observed features produced by MS (e.g., the presence of a DWR knee with a large vertical extent).

The evidence presented here should eliminate any doubt that GPM must account for MS in deep convection. However, MS effects can be more subtle and must be carefully investigated by GPM algorithm developers. Based on preliminary analysis of a limited number of cases, it appears that, as first rule of thumb, Ka profiles exceeding the 30 dB*Z* level in the ice phase below −5^∘^C cannot be safely studied with single‐scattering approximation. The 30 dB*Z* level is suggested here as a first‐order proxy for the presence of significant amounts of high‐density ice particles, and the −5^∘^C limit is only to exclude the layer where aggregation of low‐density ice or melting snowflakes can generate reflectivity in excess of 30 dB*Z* but with no significant multiple scattering. A more qualitative yet arguably more correct statement is that profiles containing significant amounts of graupel or hail cannot be safely studied with single‐scattering approximation. Ongoing work aims at developing a flagging criterion for MS‐contaminated events and at establishing an operational retrieval for the ice microphysical characterization in convective towers.
